# BoxesZero: An Efficient and Computationally Frugal Dots-and-Boxes Agent

**DOI:** 10.3390/e27030285

**Published:** 2025-03-09

**Authors:** Xuefen Niu, Qirui Liu, Wei Chen, Yujiao Zheng, Zhanggen Jin

**Affiliations:** 1 School of Computer and Communication Engineering, Northeastern University, Qinhuangdao 066004, China; 2 School of Computer Science and Engineering, Northeastern University, Qinhuangdao 066004, China

**Keywords:** decision making, deep reinforcement learning, machine learning, deep neural networks

## Abstract

In recent years, deep reinforcement learning (DRL) has made significant progress in the field of games. A prime example is AlphaZero, which, despite the formidable capabilities showcased, deters many from exploring its potential because of its demands for substantial computational resources. In this paper, we introduce BoxesZero, a computationally frugal Dots-and-Boxes agent that can achieve a high level of performance using relatively fewer computational resources. BoxesZero utilizes a novel and insightful training approach called “backward training”, which starts by training from high-reward states near the end of the game and gradually trains earlier stages of the game. It also incorporates the domain knowledge of Dots-and-Boxes, such as endgame theorems, to accelerate the Monte Carlo Tree Search (MCTS) process. Furthermore, we extend the existing endgame theorems (which only include long chains) to encompass scenarios with 1-chains and 2-chains, providing corresponding proofs, which we refer to as the extended endgame theorems. This novel agent, BoxesZero, can achieve a high level of playing strength much faster than AlphaZero, substantially improving the model’s learning efficiency. With carefully tuned parameters and limited GPU resources, BoxesZero surpasses the strongest open-source Boxes agents, PRsboxes and DabbleBoxes. Experimental results demonstrate that BoxesZero achieves an ELO rating comparable to AlphaZero in significantly less time. Furthermore, BoxesZero won the championship in the Dots-and-Boxes category of the 2024 Chinese Computer Game Competition.

## 1. Introduction

Artificial intelligence has made significant strides in the field of board games, with numerous classical reinforcement learning (RL) algorithms, such as Q-learning [1], DQN [2], PPO [3], and SAC [4], achieving state-of-the-art results. However, when faced with complex strategic decision-making problems that feature vast state spaces and delayed rewards, these agents often underperform, still grappling with challenges like data sparsity and low utilization. It was not until the advent of AlphaGo [5] in 2016 that the immense potential of deep reinforcement learning(DRL) in board games was fully unveiled. Since then, a series of exceptional algorithms have emerged, including AlphaGo Zero [6], AlphaZero [7], KataGo [8], and MuZero [9]. These algorithms ingeniously integrate deep neural networks with Monte Carlo Tree Search (MCTS) [10,11], demonstrating superhuman performance in classic board games like Go, chess [12,13], and shogi [14]. In 2017, the Transformer [15,16] architecture showcased its remarkable potential in fields such as Natural Language Processing (NLP) and Computer Vision (CV) owing to its ability to efficiently capture long-range dependencies in sequential data. Consequently, researchers began exploring the integration of this multi-head attention mechanism into RL, where these architectures have shown great potential in enhancing game efficiency and optimizing decision-making processes [17,18]. Nevertheless, the aforementioned methods demand substantial GPU computational power, making them inaccessible to many researchers. We are committed to developing an agent that efficiently utilizes computational resources, reduces computational power requirements, and possesses high-level gameplay abilities.

Dots-and-Boxes (Boxes) [19] is a classic combinatorial game with unique rules and a complex strategy space and provides an ideal platform for validating and developing new algorithms. Its fairness ensures that the final score depends solely on the players’ strategic choices, making it a valuable experimental environment for exploring new algorithms. Although 4×5 Boxes has been solved [20], the game’s complexity increases exponentially with the board size, rendering this approach impractical for larger boards. The diversity and challenging nature of strategies in the opening, middle game, and endgame phases demand substantial computational resources from traditional reinforcement learning and search methods, making them difficult to apply effectively. Currently, approaches for 6×6 or larger boards are primarily dominated by traditional search methods (such as Alpha-beta [21]) combined with handcrafted evaluation functions and DRL methods, which require extensive computational resources. Furthermore, Boxes often require progressing to the very end to determine the winner, leading to a severe sparse reward [22] problem. Therefore, we chose Boxes to test our architecture because it is suitable for games with long time horizons and well-defined termination states.

To address the aforementioned issues of sparse rewards and high computational resource demands, we introduce a novel self-play training architecture called backward training. Our model begins learning from the states near the end of the game and gradually progresses as it learns the appropriate values for the states approaching termination. During this process, we leverage certain conditions to extract high-value nodes from MCTS for training to mitigate the challenges posed by data scarcity, primarily focusing on training the value network. Upon reaching the initial state, we cease extracting nodes from the tree and commence comprehensive reinforcement training for the policy network. Additionally, we introduce pruning and acceleration techniques for MCTS and extend the endgame theorem [23] to include cases with 1-chains and 2-chains, providing proofs for these extended endgame theorems.

To recap, the core innovations incorporated into BoxesZero are as follows:

- **Addressing the Sparse Reward Problem**: In traditional RL, agents often require multiple steps to obtain a reward, which increases the difficulty of learning. Our backward training method prioritizes learning the values of states near the end of the game and gradually progresses forward, significantly mitigating the sparse reward problem.
- **Mitigating Data Scarcity**: Typically, in traditional MCTS, only data from the root node are used for training, which may overlook high-quality nodes and necessitate a large number of simulations, thereby wasting computational resources. To address this, we introduce a constraint mechanism to filter out frequently visited high-quality nodes in MCTS for training. Experiments show that this method greatly improves the learning efficiency of the agent’s value network during the early stages of training. However, during the reinforcement training phase, we eliminate this data extraction method in the tree to enhance the policy network.
- **Incorporating Domain Knowledge**: In reinforcement learning, effectively utilizing prior knowledge without hindering exploration embodies the balance between exploration [24] and exploitation, which is a significant challenge. In our MCTS process, we introduce a bias constraint to balance exploration with the avoidance of unreasonable moves, focusing computational resources on common game states and improving efficiency. Additionally, we utilize forced endings (endgame theorems) to accelerate MCTS and enable it to obtain more accurate rewards.
- **Solving Endgames with 1-Chains and 2-Chains**: The existing endgame theorem for Boxes only applies to cases with long chains (*length*≥3) and long loops (*length* ≥4). However, in actual gameplay, cases with 1-chains and 2-chains are very common. Therefore, we provide endgame theorems and proofs involving cases with 1-chains and 2-chains. This extension significantly improves the hit rate of endgame situations in MCTS.

## 2. Related Work

Two primary categories of methods dominate the current landscape of game-playing artificial intelligence: one is represented by AlphaZero, which combines DRL with MCTS or Enhanced Monte Carlo Tree Search(EMCTS) [25]; the other is represented by traditional search methods, such as MiniMax [26], Proof Number Search (PNS) [27], Principal Variation Search (PVS) [28], Rapid Action Value Estimation (RAVE) [29], and others.

### 2.1. Deep Reinforcement Learning Algorithm for Boxes

**AlphaZero** [7] is an algorithm that has achieved remarkable success in DRL. By integrating self-play with deep neural networks and MCTS, AlphaZero has learned complex strategies without relying on human expert knowledge. Its innovative approach represents a significant evolution in the application of deep reinforcement learning, particularly in strategic games.

The combination of DRL and MCTS can be observed across various gaming environments, having a profound impact. DRL allows agents to learn optimal policies through interactions with their environment, while MCTS efficiently explores the decision space, balancing the exploration of new strategies with the exploitation of known successful ones. This combination empowers systems like AlphaZero to go beyond traditional methods, making substantial advancements in gameplay complexity and strategy development. One of AlphaZero’s most notable achievements is its ability to defeat top human players and established algorithmic programs in games such as Go, chess, and shogi. In the game of Go specifically, AlphaZero not only broke the long-standing dominance of human players but also discovered many innovative strategies through self-play, some of which had never been seen before by humans. This illustrates AlphaZero’s powerful learning capabilities and its potential to address complex decision-making problems in various domains.

AlphaZero utilizes a deep neural network parameterized by θ, defined as $\left(p,v\right)=f_{\theta }\left(s\right)$. For any given board position *s*, the network generates an action probability $p_{a}=P_{r}\left(a|s\right)$ for each possible action *a*, along with a state value *v*. This combination of (p,v) is used to guide the MCTS in selecting actions. The strategy π returned by MCTS is then used to train the policy network, while the game’s final reward *z* is utilized to train the value network, i.e., (p,v)→(π,z). Unlike standard MCTS implementations, AlphaZero’s MCTS process specifically consists of four core steps:

1. **Selection**: During the selection phase, the algorithm begins at the root node and utilizes the Upper Confidence Bound for Trees (UCT) [30,31] formula to select child nodes along an already expanded path until it encounters a non-expanded node. The UCT formula balances exploration and exploitation by selecting the optimal child node. It is expressed mathematically as follows:
  (1) $$UCT\left(s,a\right)=Q\left(s,a\right)+C_{puct}\cdot P\left(s,a\right)\cdot \frac{\sqrt{N(s)}}{1+N(s,a)}$$
  where Q(s,a) represents the average value of the action taken at state *s*, P(s,a) denotes the prior probability of taking action *a* in state *s* (as provided by the neural network), N(s) is the visit count for state *s*, N(s,a) is the visit count for the action a in state *s*, and $C_{puct}$ is a constant parameter that determines the exploration factor. This formula facilitates a trade-off between exploring less-visited paths and exploiting those believed to yield high rewards.
2. **Expansion**: Upon reaching a non-expanded node during the selection phase, the expansion phase commences. At this stage, the search tree is augmented by adding unexplored child nodes, thus increasing the search space and allowing for a more comprehensive exploration of possible actions.
3. **Evaluation**: At the newly added nodes, the neural network evaluates the current state and generates two outputs: the policy vector *p* (indicating the probabilities of each action) and the value *v* (representing the expected outcome from that state). This evaluation informs future decision-making processes and helps refine the strategy.
4. **Backpropagation**: After the evaluation step, the results are backpropagated from the current node to the root node along the path traversed during selection. The search counts and state value *v* for each node are updated based on the propagated results. This mechanism reflects the win rates or returns of the current strategy, effectively informing the model about the success of actions taken during the simulation.

Attempts to apply the AlphaZero framework to boxes have yielded less satisfactory results. The Dots-and-Boxes game always requires that you fill in all possible moves before it ends, which leads to the problem of sparse reward signals. In particular, in the early stages of the game, rewards are difficult to propagate back to the root node, which means that a large amount of data is required to train the neural network effectively. As a result, AlphaZero’s approach requires a long time to develop a reasonable level of play, and the computational resource demands are often prohibitive for most users, leading to less noticeable replication results.

### 2.2. Traditional Search Algorithm for Boxes

**MiniMax** [26] is one of the earliest and most widely applied algorithms in game theory, particularly suited for perfect information games like Boxes. The MiniMax algorithm traverses the game tree to minimize the potential loss in the worst-case scenario. When applied to Boxes, MiniMax is typically combined with the Alpha-Beta pruning technique to enhance computational efficiency by reducing the number of nodes that need to be evaluated. However, as the complexity of the game increases, the performance of MiniMax degrades significantly due to the exponential growth of nodes in the game tree, which is especially evident in strategy-intensive games like Boxes. Nevertheless, MiniMax remains a valuable reference tool in the research and development of more advanced game algorithms. In the study by Joseph K. Barker and Richard E. Korf, titled “Solving 4×5 Dots-and-Boxes” [23], they proposed a solution based on the MiniMax algorithm. By constructing a complete game tree and combining Alpha-Beta pruning to enhance computational efficiency, they also effectively utilized board symmetry, applied optimization strategies for the midgame and endgame, and used transposition tables. The combination of these techniques allowed them to successfully solve the 4×5 size Boxes game. However, as the size of the board increases, the method becomes less applicable, so the largest fully solved size of Boxes remains 4×5.

## 3. Game of Dots-and-Boxes

This section will introduce the fundamental rules of the Dots-and-Boxes game, along with key strategic principles and the endgame theorems, to build a comprehensive understanding of the game.

### 3.1. Basic Rules of Boxes

Boxes is a classic combinatorial game that enjoys widespread popularity across the world. The game is played on a rectangular grid of dots, where players take turns connecting two adjacent dots to form a line segment. Whenever a player completes the fourth side of a box, the player claims that box and gets to take another turn. The objective of the game is to complete more boxes than the opponent, and the player who secures the most boxes by the end of the game wins. Although the rules of the game are simple, the strategies involved are very complex, especially in the endgame phase. Fortunately, there are endgame theorems.

**Figure 1:**
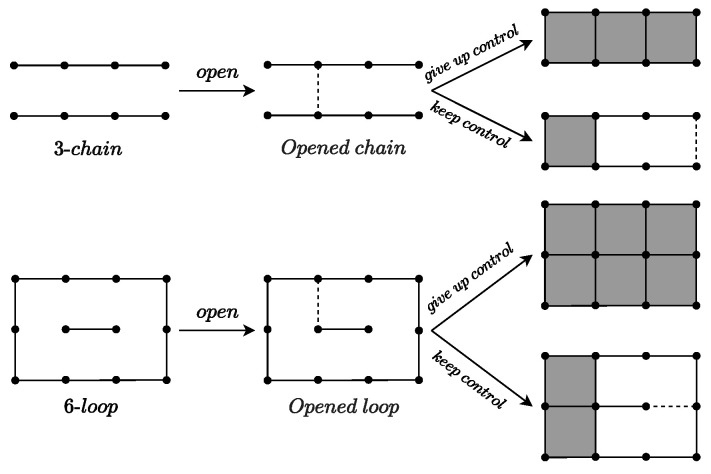
The figure shows chain and opened chain, loop and opened loop, as well as two strategies for after opening, keep control and give up control, which correspond to a 3-chain and a 6-loop, respectively.

To better introduce the endgame theorems, we first present several special structures in Dots-and-Boxes: chain and opened chain, loop and opened loop, as shown in Figure 1. The terms “chain” and “loop” are named after their shapes. They share a common characteristic: If any move is made within a chain or loop, the opponent can claim all the boxes within it. For an opened chain (length≥3) or an opened loop, there are generally two strategic options:

1. Capture all the boxes in the chain or loop.
2. Capture part of the chain (all but two boxes) or part of the loop (all but four boxes), leaving the remaining boxes for your opponent.

Option 1 is referred to as “*give up control*” because occupying all the boxes may require the player to make an additional move. This move could potentially open up another chain or loop, thereby transferring the choice to the opponent and placing the player in a passive position. Option 2 is termed “*keep control*” because if the opponent occupies the remaining squares, they must also make another move, allowing us to maintain control over the remaining positions. Choosing between “*keep control*” and “*give up control*” for chains or loops involves combinatorial game theory. The two strategies mentioned above are exhibited in Figure 1. In the figure, the “open” operation refers to making a move in a chain or loop.

**Figure 2:**
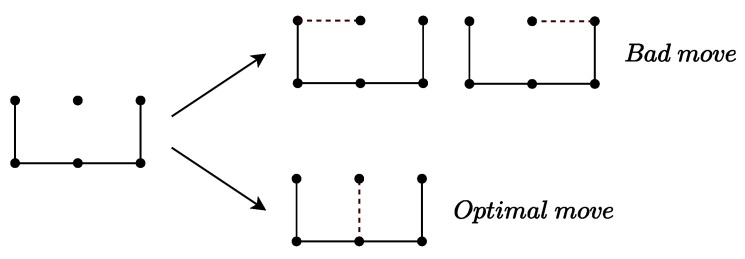
Optimal strategy for capturing a 2-chain.

Next, we introduce three commonly used strategies in Boxes, which will lay the foundation for applying endgame theorems and domain knowledge to accelerate the tree search in Section 4.5:

(1) **2-chain strategy:** When you need to open a 2-chain, you should always select the middle edge, the one between the other two edges. If you choose either of the other two edges, your opponent has two options: keep control or give up control. However, if you choose the middle edge, your opponent only has one option: give up control. Clearly, choosing the middle edge limits the choices of your opponent, placing them in a more disadvantageous position. Therefore, when playing against an intelligent opponent, selecting the middle edge is the optimal strategy, as illustrated in Figure 2.

(2) **Equivalent Edges:** In a chain (length≥3) or a loop (length≥4), all edges are equivalent. This is because, regardless of which edge is played in a chain, the opponent faces two choices: keep control or give up control. In each case, given the same choice, the outcome in terms of boxes secured will ultimately be the same. For instance, in a 3-chain, if a player moves along any of the four edges, and if the opponent chooses to keep control, the first player will secure two boxes while the opponent secures one box. Conversely, if the opponent chooses to give up control, they will take all the boxes.

(3) **Forced Move Strategy:** For an opened 1-chain, always take the box and make an additional move. If you do not take the box and instead make a move elsewhere, your opponent can take the box and then make another move on the remaining board. Therefore, capturing this box is the optimal strategy. If the middle edge of a 2-chain is opened, you should capture the entire 2-chain (equivalent to two opened 1-chains). For a chain of length≥3, take (length−2) boxes. If a loop of length≥4 is opened, you should take (length−4) boxes.

### 3.2. Endgame Theorems

This subsection offers a concise overview of the content related to the endgame theorems presented in the paper [23]. We provide a simple definition for the endgame: a position consisting solely of long chains (length≥3) and long loops (length≥4), where each box has either 2 or 4 edges occupied. For simplicity, we will collectively refer to the long chain as a chain. Next, we will provide definitions for the terms “opener” and “controller” in the context of the endgame.

**Opener:** In your turn during the endgame, you need to choose a loop or a chain to open. The player making this choice is called the opener.

**Controller:** Once the opener selects a component *C* (loop or chain), the controller, who is the other player, then has two options: keep control or give up control.

**Figure 3:**
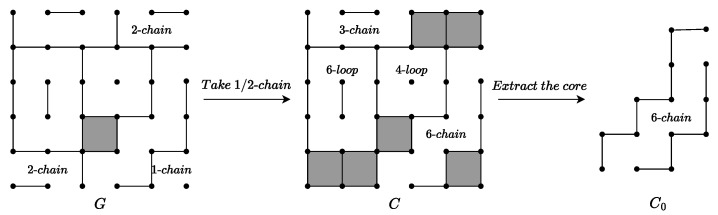
Example of the endgame theorem’s application.

Before delving into the endgame theorems, we define a board position as *G*. For instance, $G=4_{l}+6_{l}+3+2^{2}+1+6$ indicates that *G* consists of a 4-loop, a 6-loop, a 3-chain, two 2-chains, a 1-chain, and a 6-chain, as well as possibly some already-claimed boxes, as shown in Figure 3.The value v(G) of any endgame *G* refers to the margin by which the controller will defeat the opener, assuming both players enter the endgame with a tied score and play optimally. Table 1 presents the definitions of the symbols and functions, all of which are defined in relation to *G*. The controlled value c(G) is defined to compute v(G), with its explicit formula given below:

(2) c(G)=size(G)−4×(#longchains)−8×(#loops)+tb(G)

where # represents the number of the component, and tb(G), which denotes the terminal bonus of *G*, is defined as follows:

(3) $$tb\left(G\right)=\left\{\begin{array}{cc} 0 & \mathrm{if}\;G\;\mathrm{is}\;\mathrm{empty}\\ 8 & \mathrm{if}\;G=(\mathrm{one}\;\mathrm{or}\;\mathrm{more}\;\mathrm{loops})\\ 6 & \mathrm{if}\;G=(\mathrm{one}\;\mathrm{or}\;\mathrm{more}\;\mathrm{loops})+(\mathrm{one}\;\mathrm{or}\;\mathrm{more}\;3-\mathrm{chains})\\ 4 & \mathrm{otherwise} \end{array}\right.$$

Following that, we will present three theorems that can be directly applied to the previously defined endgame scenarios. These theorems offer insight into the number of boxes that can be won or lost in the endgame and the optimal next moves.

Theorem 1 provides the opener strategy for endgames, Theorem 2 offers the controller strategy, and Theorem 3 explains how to calculate the value of an endgame, denoted as *v*. By applying these three theorems, once the game reaches an endgame position, all subsequent moves can be determined entirely by the theorems. Additionally, the number of boxes won or lost can be precisely calculated, allowing for an immediate assessment of the game’s outcome upon entering the endgame phase. The three theorems are as follows:

**Theorem** **1.** *(Opener Strategy) Suppose G is a nonempty Dots-and-Boxes position that consists of loops and long chains. In each of the following cases, opening the shortest loop is optimal:*

*1.*  *c(G)≥ 2 and G= 3+ (one or more loops);*
*2.*  *c(G)∈{0, ±1} and G= 4_l_ + (anything except 3 + 3 + 3);*
*3.*  *c(G)≤−2 and G= 4_l_ + 3 +H, where 4 ∣size(H) and H has no 3-chains.*

*In all other cases, the standard move is optimal.*

**Theorem** **2.** *(Controller Strategy) If the opener has just opened a component C of G, the following gives an optimal move to the controller. Keep control if C is a loop and c(G−C)> 4, or if C is a chain and v(G−C)> 2; otherwise, give up control.*

**Theorem** **3.** *(Values Procedural) Suppose G is a nonempty Dots-and-Boxes position consisting of loops and long chains. Then*

*1.*  *if c(G)≥ 2, then v(G)=c(G);*
*2.*  *if c(G)= 0, G has a 4-loop, and G≠ 4_l_ + 3 + 3, then v(G)= 0;*
*3.*  *if θ(G)= 0, or if θ(G)= 1 and size(G)≡ 3mod4, then*
(4) $$v=\Phi^{f(G)}\left(\Theta^{\theta (G)}\left(\Sigma^{s(G)}\left(c\left(G_{0}\right)\right)\right)\right)$$

*In all other cases, v= 1 or 2 according to the parity of G.*

$G_{0}$ is a subset component of *G*. It includes the following components of *G*: its loops consist of all loops of length≥8, and its chains are either all chains of length≥4 (if any exist) or the 3-chain (if *G* contains one and no other chains). Furthermore, the symbol $\theta^{\prime }$ denotes the number of 3-chains in $G-G_{0}$. The functions Σ, Θ, and Φ are defined as follows: Σ(x)=|x−4|+2, Θ(x)=x−1, and Φ(x)=|x−4|. In the presented formula, $\Phi^{f(G)}$ indicates that the Φ operation is applied iteratively f(G) times, where f(G) corresponds to the number of 4-loops within G. Likewise, $\Theta^{\theta (G)}$ and $\Sigma^{s(G)}$ denote the iterative application of the Θ and Σ operations θ(G) and s(G) times, respectively.

## 4. Methodology

In this section, we will detail the key techniques employed in BoxesZero, including the backward training architecture, the extension of the endgame theorem, data augmentation, and MCTS pruning.

### 4.1. Reinforcement Learning in BoxesZero

In BoxesZero, we also use a deep neural network $f_{\theta }$ with parameters θ, which takes the multi-channel representation of the board state *s* as input and outputs an action probability *p* and a state value estimate *v*, i.e., $\left(p,v\right)=f_{\theta }\left(s\right)$. In the MCTS search, each node *s* stores a visit count N(s) and a state value Q(s), while each edge (s,a) maintains a visit count N(s,a) and an action value $Q\left(s,a\right)=\frac{1}{N(s,a)}\sum_{s^{\prime }|s,a\to s^{\prime }}v\left(s^{\prime }\right)$. Here, the notation $s,a\to s^{\prime }$ indicates that after taking move *a* from position *s*, the simulation eventually reaches state $s^{\prime }$. After that, we can calculate the state value of node Q(s) using the following formula:

(5) $$Q\left(s\right)=\frac{\sum_{a}N\left(s,a\right)\cdot Q\left(s,a\right)}{\sum_{a}N\left(s,a\right)}$$

The *Q* referred to in the following discussion denotes the Q(s) output by MCTS, while *v* is the value output by the network.

In training the policy network, we use the action probabilities π provided by MCTS, which is consistent with the approach used by AlphaZero. At the same time, we employ a weighted combination of the *Q* value from the root node of the tree and the final reward *z* (which belongs to {1, −1}) as the reward for training the value network. This differs from AlphaZero’s method, which solely uses the final reward *z*. We do this primarily for the following reasons:

Firstly, assigning the same reward *z* to all states can introduce significant bias. While *z* accurately reflects the value of the last few moves, it may overlook earlier steps due to the inherent randomness in the final action selection by MCTS. If a particular choice leads to the loss of the entire game because of random factors, it can negate the value of all previous steps, which is clearly inefficient. This limitation requires the model to consume more computational resources during training.

Secondly, while *z* provides a binary classification of outcomes (win or loss), it lacks the detailed representation of state values necessary in complex game scenarios. Different states may correspond to varying probabilities of winning, but *z* fails to capture the subtle differences in value between these states. The diversity and complexity of intermediate positions highlight that relying solely on the final win-loss outcome does not fully reflect the true potential and value of these states.

On the other hand, the value *Q* from MCTS offers a nuanced estimation of the expected value based on the current state and moves taken, allowing for better decision-making throughout the game. However, it may be influenced by noise and estimation biases inherent in the MCTS process. Therefore, combining the final reward *z* with the *Q* values provides a more robust approach. This combination allows the model to leverage the clear win-loss signal from *z* to learn long-term outcomes, while also incorporating the detailed state evaluations provided by *Q*. By doing so, the value network can better understand the overall strategy and make informed decisions, ultimately improving performance and reducing uncertainty in the learning process.

Data collected through a new reinforcement learning algorithm, backward self-play (detailed in Section 4.2.1), is used to train and update the neural network’s parameters θ.

### 4.2. Overview of Backward Training

We introduce a novel training methodology called backward training. We divide the backward training process into two key stages: the value network reinforcement stage and the policy network reinforcement stage. The value network reinforcement stage sets a step limit and utilizes the data generated by the model after exceeding this limit for training. Once the model satisfies a predefined advancement condition, we propel the model forward. The policy network reinforcement stage commences after the model advances to step 0. At this point, we utilize data from all steps for training, with the primary objective of achieving convergence for the policy network. We will now elaborate on the backward self-play method and elucidate the model advancement conditions.

#### 4.2.1. Backward Self-Play

During the initial training phase, AlphaZero typically uses extensive self-play to collect data for training. However, due to the model’s weak playing strength in the early stages, only the data from the last few steps are relatively accurate, while the data from the initial steps is mostly randomly generated. This leads to inconsistent training data quality, affecting the network’s convergence speed and hindering the improvement of the model’s capabilities.

To mitigate this issue, we propose an improvement to the traditional self-play paradigm, aiming to fully utilize computational resources and enable the model to achieve a high level of proficiency within a shorter timeframe. Specifically, during the initial stages of model training, we selectively learn from the data, focusing only on the last few steps that are closer to the final reward and ignoring the lower-quality data from the earlier steps. As training progresses, the model’s predictive ability gradually strengthens, and the accuracy of the data from the later steps improves accordingly. At this point, we gradually incorporate data from earlier steps into the learning process, eventually encompassing all steps.

We define a threshold, “start searching” (abbreviated as st), to determine when to begin using MCTS. The entire self-play process can be divided into two stages. The first stage involves collecting data from the end to the beginning, utilizing the *Q* values returned by MCTS to train the value network. During this phase, the value network converges relatively quickly. As the number of training iterations and time increase, the model’s predictive capability is enhanced, which causes the st value to progress forward until it reaches step 0. At this point, we enter Stage 2, where we start collecting data anew for self-play training and use the outcome *z* to assist in refining the value network. The introduction of the *z* at this juncture facilitates the convergence of the global strategy. The reward training value network is derived by combining the *z* and *Q* with weights of 0.75 and 0.25, respectively. This particular 0.75/0.25 weighting has been empirically established as effective and is widely adopted in machine game-play scenarios.

For the move selection before the st step, we employ a greedy handcrafted policy in the game of Boxes. The core idea of this policy is to minimize the opportunities for the opponent to score. The policy prioritizes filling the fourth edge of a box, followed by the first/second edges and, lastly, the third edge. In practice, most Boxes models follow this greedy strategy. However, some advanced models might intentionally leave some boxes open in the midgame to create a more favorable endgame for themselves. Since we do not learn the knowledge before st, this greedy strategy does not negatively impact the model’s improvement. Moreover, the game states generated by this greedy strategy contain numerous chains and loops, which are similar to our definition of the endgame. This allows the model to encounter more endgame scenarios during MCTS, thereby enhancing its learning efficiency in the endgame phase. For other board games, if there are no applicable hand-made rules or strategies in the early stages, we can directly use the smoothed policy network values *p* to select moves. Furthermore, the game states generated using the above strategy are not searched; therefore, data sampling is not performed.

**Figure 4:**
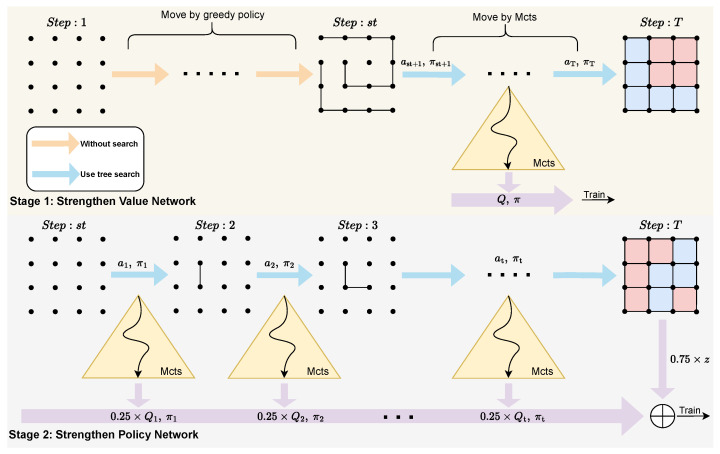
The self-play process comprises two phases. The first phase involves a backward pass, emphasizing the training of the value network. The second phase utilizes a forward pass, incorporating *z* to finalize the policy network.

After reaching st, each subsequent move is determined using MCTS until the game ends. The backward self-play process is illustrated in Figure 4.

#### 4.2.2. Model Advancement Conditions

In board games, the complexity of the state of the game is typically lower in the early and late stages, whereas the midgame is the most complex. As a result, more iterations may be required during the midgame, while fewer iterations are needed in the late game. Therefore, determining whether the current step is sufficiently trained and whether it is time to move forward is critical.

We evaluate the training sufficiency of a step by statistically analyzing *Q*-value variations observed across multiple self-play game states within a single iteration. Notably, we observed that as training progresses, |Q| output by the MCTS gradually increases from 0 to 1. This is because, when the policy network is weak, the quality of nodes explored by MCTS is poor, making it difficult for *Q* to accurately reflect the expected outcome. In such cases, *Q* in the search tree tend to cancel each other out, as some nodes predict win and others predict lose, resulting in an average *Q* close to 0. As training continues and the policy network improves, MCTS is able to identify favorable nodes, and |Q| approaches 1, reflecting clearer expectations of the results. Therefore, the proximity of *Q* of the root to 1 can serve as an indicator of training progress.

In each iteration, we assess the neural network’s performance by calculating two proportions: nvr and mqr. nvr represents the fraction of states that satisfy both v×Q≥0 and |v|≥nvth. mqr is the proportion of positions where |Q|≥mqth. (Parameters mqth, nvth, nvr, and mqr are described in Table 2.) The condition v×Q≥0, where v is the neural network’s prediction and Q is a more precise value, measures the network’s ability to predict the correct sign. The additional condition |v|≥nvth further refines this measurement by filtering out predictions with low confidence. Finally, the relationship between |v| and 1 is used as an indicator of the neural network’s overall capability in outputting realistic values. If both nvr and mqr are larger, it indicates that our model performs better at distinguishing winning and losing positions.

Ideally, |Q| for each step would reach 1. However, in practical observations, without sufficient computational resources, it is difficult to achieve such values for the first 20 moves. If training continues for an extended period without meeting strict conditions and remains stuck in one step, it can lead to overfitting of the network. Since later steps will continue to be trained after moving forward, we need to set a maximum number of iterations that satisfy the progression conditions to prevent being stuck at a particular step.

Based on the analysis above, we present the following advancement conditions.

**Advancement Conditions:** The conditions for progressing consist of two criteria: a constraint on *Q* and *v* and a constraint on the number of iterations. If either of the two conditions is met, the model can advance. The specific conditions are as follows: (1) currentiter−lastiter≥mit. (2) mqr≥mqrthandnvr≥nvrth.

The “current iter” refers to the current iteration number of the model, while “last iter” indicates the iteration number where the model was during its last advancement (where one iteration refers to generating data through multiple self-play games for training). “mit” denotes the maximum iteration threshold for model advancement. The specific meanings of the variables “mqr”, “mqrth”, “nvr”, “nvrth”, and “mit” are provided in Table 2. In condition (2), all the variables are used to calculate the ratio of self-play game states that satisfy *Q* and *v* conditions within one iteration, which determines whether the model can advance.

At the end of each iteration, the model checks if the advancement conditions are met. If they are, advance the model by β steps.

### 4.3. Data Augmentation

The utilization of a backward training strategy facilitates accelerated convergence to accurate reward estimations, leading to higher training data efficacy. This approach results in a significantly increased prevalence of high-value nodes within the search tree. Consequently, we implement conditional selection mechanisms within Monte Carlo Tree Search to strategically select such nodes for further training, thereby addressing the inherent data scarcity challenges in reinforcement learning.

As states approach the end of the game, they are closer to receiving rewards, causing the value of |Q| returned by MCTS to approach 1. Conversely, for states that are further from the end, reward propagation becomes more difficult, resulting in |Q| approaching 0. Therefore, we can assume that nodes with larger |Q| have higher quality, indicating that the network can clearly distinguish the win or loss of the state. We use this characteristic as one of the criteria for evaluating high-quality nodes. Relying solely on *Q* constraints is insufficient to determine the quality of a node. Therefore, we also incorporate a weak constraint based on the visit count *N* of the tree node.

**Figure 5:**
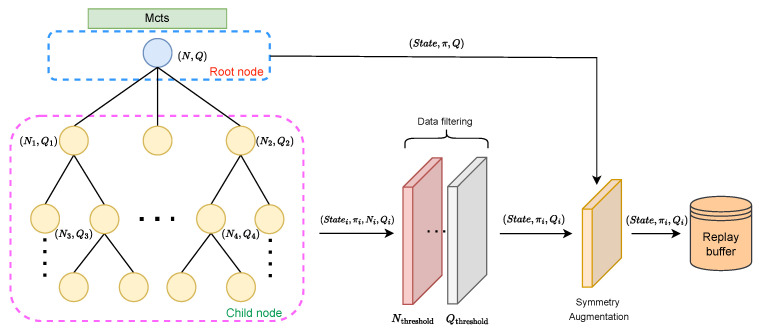
The process of data collection from MCTS, enhanced by data augmentation techniques.

We impose higher (N,Q) constraints for states closer to the end and lower constraints for states further to the end. Due to prior training, states closer to the game’s end exhibit higher quality. To maintain a balanced data distribution across different steps, these nodes require stricter selection criteria. The data augmentation method is illustrated in Figure 5, and the node selection criteria are as follows: $N>N_{\mathrm{threshold}}$ and $Q>Q_{\mathrm{threshold}}$. The specific settings for $N_{\mathrm{threshold}}$ and $Q_{\mathrm{threshold}}$ are provided in Table A2. After filtering the data, we applied rotational symmetry augmentation to further expand the dataset before storing it in the experience replay buffer.

During the value network reinforcement stage, we emphasize the use of this data augmentation method, as it can accelerate the convergence of the value network. However, we do not employ data augmentation during the policy network reinforcement stage. This is because the augmented data have smaller *N*, which can interfere with the convergence of the policy network.

### 4.4. Improved Endgame Theorems

The previous endgame theorem restricts its application to positions involving long chains, making it inapplicable to positions that include 1-chain and 2-chain. However, in high-level gameplay, 1-chain and 2-chain are very common. To address this, we extended the endgame theorem to apply to positions that include 1-chain and 2-chain. We proposed an open strategy and provided a formula for calculating the value. After extensive validation on positions involving 1-chains and 2-chains, we confirmed the correctness of the extended theorem.

**Theorem** **4.** *(Extended Opener Strategy) Suppose G consists of chains and loops and that there exists a 1-chain or a 2-chain. If there is at least one 1-chain, opening a 1-chain is optimal. If there is no 1-chain but a 2-chain exists, opening the 2-chain and following the 2-chain policy is optimal.*

**Theorem** **5.** *(Extended Values) Suppose G consists of chains and loops, containing k 1-chains and n 2-chains. Let C represent the remaining portion after removing all the 1-chains and 2-chains. The value v(C) can be determined using Theorem 3. Then, the value v(G) is given by the following:*

*1.*  *If k is odd and n is odd: v(G)=v(C)− 1;*
*2.*  *If k is odd and n is even: v(G)= 1 −v(C);*
*3.*  *If k is even and n is odd: v(G)= 2 −v(C);*
*4.*  *If k is even and n is even: v(G)=v(C).*

Since 1-chain and 2-chain (because of the 2-chain strategy) do not involve control strategies, there is no need to consider the control policy. The proof of the theorem is provided in Appendix C.

Figure 3 illustrates an example of the application of the theorem: $G=4_{l}+6_{l}+3+2^{2}+1+6$, we want to calculate v(G). According to Theorem 5 and with k=1 and n=2, we have v(G)=1−v(C). By applying Theorem 4, which requires occupying all 1-chains and 2-chains, we obtain $C=4_{l}+6_{l}+3+6$. We then calculate v(C) using Theorem 3, as θ(G)=1 and size(C)=19≡3(mod4), so we choose the third case of the theorem. First, based on the definition of $G_{0}$, we find $C_{0}=6$. According to Formula (2), we have $c(C_{0})=6$. Given s(C)=1, f(C)=1, and θ(C)=1, we calculate v(C) as follows: $v\left(C\right)=\left|\left(\left|c(C_{0})-4\right|+2-1\right)-4\right|=1$. Therefore, v(G)=0.

### 4.5. Pruning in MCTS

Based on the four core steps of MCTS outlined in Section 2.1, we prune the search tree that incorporates the endgame theorems and the three strategies described in Section 3.1. This approach reduces ineffective searches in MCTS by introducing endgame theorems and chain-and-loop strategies during the expansion phase, allowing for early win/loss predictions. This enables rewards to be quickly propagated from child nodes to the root, thereby accelerating the convergence of both the policy and value networks. The specific details are as follows:

1. **Endgame Theorem Pruning**: During the search process, if a node’s position *s* meets the definition of an endgame, Theorems 3 and 5 can be directly applied to calculate the value v(s). Taking into account the current territory situation, we can calculate the score of state *s* as follows: scores=currentboxes−opponentboxes−v(s). The positive and negative values of scores correspond to Q(s)=±1 in the MCTS nodes, thus achieving the purpose of pruning. This allows the search tree to encounter actual rewards earlier. It has been verified that for a 6×6 Dots-and-Boxes game, which consists of 60 moves in total, MCTS can start detecting endgames around the 30th move, enabling effective reward propagation.
2. **Chain-Loop Pruning**: In the tree, if the current position contains an opened chain or opened loop, the forced-move strategy mentioned in Section 3.1 can be applied to execute multiple consecutive moves, thereby reducing the search depth of MCTS. This leads to faster encounters with the endgame, thus mitigating the issue of sparse rewards and significantly improving search efficiency and decision quality.

## 5. Implementation Details

### 5.1. System Overview

We primarily adopt a distributed system architecture, which is divided into three main components: the Computing Center, the Controlling Center, and the Training Center. The Computing Center consists of ten computers, each equipped with an Intel(R) Core(TM) i5-8500 CPU. The Controlling Center and Training Center each consist of one machine equipped with an NVIDIA GeForce RTX 3060 GPU. The Computing Center generates self-play data, the Training Center learns from a shared experience replay buffer, and the Controlling Center manages data distribution and network parameter updates. This design enables concurrent data collection and network training, significantly improving time efficiency.

**Figure 6:**
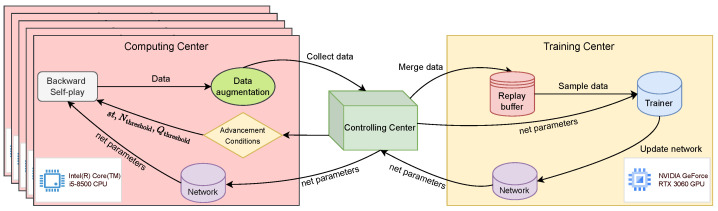
Diagram of a distributed data collection system architecture.

The system operates as follows: The Controlling Center sends data collection tasks and neural network parameters to the ten computing nodes. All computing nodes initiate self-play simultaneously and gather data on MCTS during the process. The data collected are then sent from the computing nodes to the Controlling Center. The Controlling Center aggregates the data and sends it, along with the network parameters, to the Training Center. The Training Center updates the network parameters and sends the new neural network parameters back to the Controlling Center. The Controlling Center begins to assess whether to advance the model based on the advancement conditions (see Section 4.2.2). This process continues cyclically. The architecture of the system is illustrated in Figure 6.

### 5.2. Neural Network Training Process

During each training cycle, the model samples a batch of data from the replay buffer for training. The replay buffer stores the (s,π,Q) information generated by the model during different iterations, which is subsequently utilized in training. In each batch, the model performs forward propagation through the neural network on the input data to compute $\left(\hat{p},\hat{v}\right)$ and finally computes the loss.

The loss function used in the training process consists of three components: policy loss, value loss, and regularization loss. The policy loss measures the difference between the model’s predicted policy and the target policy (i.e., the π provided by MCTS). The value loss measures the difference between the model’s predicted value and the target value (i.e., the *Q* provided by MCTS). The regularization loss mitigates overfitting by penalizing large weights of the neural network parameters. To ensure that recent data are prioritized during training, a decay factor is introduced. This factor adjusts the contribution of each sample to the loss based on its iteration count: samples closer to the current iteration contribute more significantly to the loss, while older samples have diminished impact. The loss function is defined as follows:

(6) $$L=\sum_{i=1}^{D}\left(-\pi \log\left(\hat{p}\right)+{\left(Q-\hat{v}\right)}^{2}\right)\cdot \gamma^{\left(i-1\right)}+\lambda {∥\theta ∥}^{2}$$

In this equation, *D* denotes the number of recent iterations stored in the replay buffer. The terms π and *Q* represent the true policy and value labels derived from MCTS, while $\hat{p}$ and $\hat{v}$ denote the policy and value predicted by the network. The decay factor for the data is represented by γ, θ signifies the neural network parameters, and λ is the coefficient for the regularization term $L_{2}$. The specific values of these parameters are provided in Table A1.

### 5.3. Neural Network Architecture

**Figure 7:**
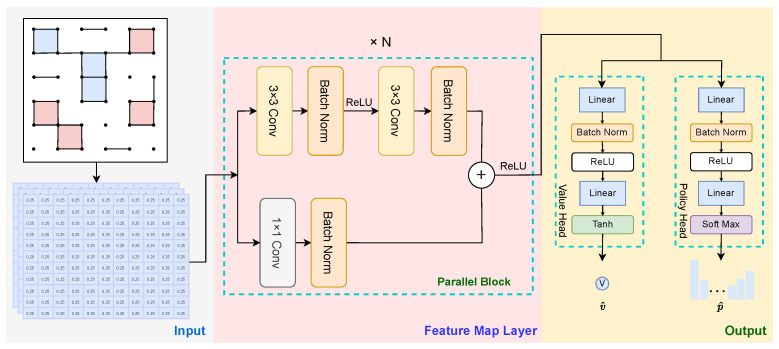
Deep network architecture for policy and value prediction in BoxesZero.

The neural network plays a crucial role in generating the estimations for the policy distribution $\hat{p}\in R^{H\times W}$ and the value $\hat{v}\in R^{1}$, effectively replacing the simulation phase of the MCTS. This network is organized into three main components: the input layer, the feature extraction layer, and the output layer, as illustrated in Figure 7.

The first part, the input layer, is responsible for the initial decomposition of the board. The network assumes that the current player is on the red side. If the player is on the blue side, the board should be flipped to switch the active player to the red side before making predictions. Therefore, there is no need to create a separate channel to represent the current player in the board state; simply perform a player conversion when inputting to the network. The original board is an H×W matrix, which we divide into three channels:

- The first channel represents the edges, where positions with edges are marked as 1, and those without edges are marked as 0.
- The second channel represents the boxes, where the positions with boxes are marked as 1, and those without boxes are marked as 0.
- The third channel represents the relative scores, calculated as follows:
  (7) scores=(numberofredboxes−numberofblueboxes)×0.25

The second part, the feature map layer, is primarily composed of parallel blocks, which further extract features from the board using Kaiming He [32] initialization to optimize the training process. Additionally, a cosine learning rate schedule, specifically through Stochastic Gradient Descent with Warm Restarts (SGDR) [33], is employed to dynamically adjust the learning rate throughout training, enhancing convergence and improving overall model performance.

The third part includes the policy head and the value head, which aggregate the features extracted by the second part and reshape them to the appropriate output.

## 6. Experiments

This section will introduce the experimental baseline, evaluation metrics, and environmental setup. We will validate the performance of the proposed pruning method, data augmentation, and backward training while also conducting an in-depth analysis of the characteristics of our model.

### 6.1. Experiment Setups

#### 6.1.1. Baselines

We evaluate our algorithm using the following well-known algorithms with established results: (1) **MiniMax [26]:** We reproduced several acceleration strategies from the paper, applying pruning and hashing techniques to the 6×6 Dots-and-Boxes. During the initial stages, when a complete search is not feasible, we utilized manual strategies for game-play. Our implementation of the alpha-beta search can achieve results in approximately 22 steps and within about 300 s. (2) **AlphaZero [7]:** We implemented the AlphaZero framework for Dots-and-Boxes, with training parameter settings as detailed in Table A1. (3) **Dabble:** (Dabble https://www.mathstat.dal.ca/~jpg/dabble/, accessed on 2 January 2025) is a computer program that uses alpha-beta search to play Dots-and-Boxes. Its understanding of the game is minimal; it knows about chains and double-crosses but nothing else. Despite this, Dabble is able to play at a reasonable skill level; higher-level strategies emerge from brute-force search. (4) **PRBoxes:** (PRBoxes https://www.dianneandpaul.net/PRsBoxes/, accessed on 2 January 2025) Uses full nimstring analysis etc.

#### 6.1.2. Metrics

We primarily use relative ELO ratings [34] and the accuracy at specific game steps to quantify algorithm performance. To compare the capabilities of different algorithms, we select models of each algorithm at various time points and conduct several matches (with alternating starting players). This allows us to compute relative ELO scores for both A and B at each time point and subsequently plot the ELO curves.

As the midgame is crucial in Dots-and-Boxes, we constructed a dataset of various game steps using the method described in Appendix E.3. This dataset allows us to test the prediction accuracy of different models at various stages of the game, thereby reflecting their respective capabilities.

#### 6.1.3. Experimental Environment

Our experimental environment is based on a distributed system that consists of three components: the computing center, the controlling center, and the training center. The computing center comprises 10 computers equipped with Intel(R) Core(TM) i5-8500 CPUs and no GPUs. In contrast, the control center and training center are represented by a single machine that features an NVIDIA GeForce RTX 3060 GPU.

### 6.2. Comparative Evaluation of Backward Training

We validate the effectiveness of the backward training method by comparing it with the traditional forward training method. We ensured that the search counts, training iterations, and learning rates were identical for both the forward model and the backward model. And neither model uses endgame theorems or acceleration methods. The key difference is that our model employs the end-to-start training method.

**Figure 8:**
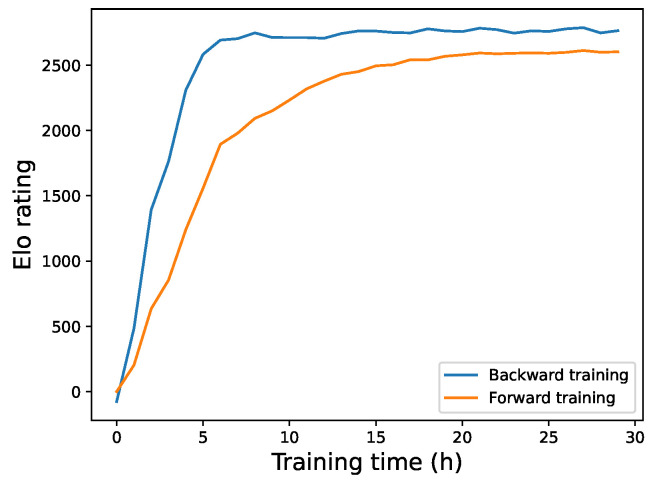
Curves of relative ELO ratings resulting from backward and forward training.

We conducted 1000 matches between models at different time points, and the resulting ELO scores over time are depicted in Figure 8. As shown in the figure, the ELO scores of the backward training show an upward trend over time, and in the early stages of training, its playing strength is significantly higher than that of the forward training. In later stages, the curve stabilizes but remains above the forward training. This indicates that backward training can achieve higher playing strength in a shorter period and demonstrates a certain level of stability. Therefore, we conclude that the end-to-start method achieves higher playing strength and enables the model to reach the same strength level in less time.

### 6.3. Analysis of Loss Function Curve

**Figure 9:**
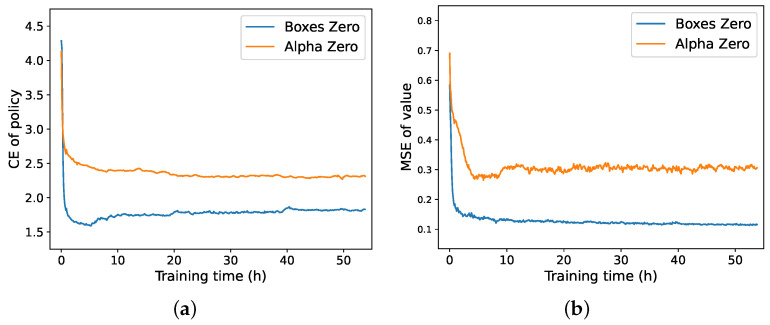
Figure (**a**,**b**) respectively show the cross-entropy loss curves of the policy network and the mean squared error curves of the value network for BoxesZero and AlphaZero after 50 h of training.

We analyzed the loss curves during the training process. Based on the loss function defined in Equation (6), we plotted the cross-entropy loss for the policy network and the mean squared error loss for the value network of AlphaZero and BoxesZero, as shown in Figure 9. From the figures, it is evident that BoxesZero exhibits a characteristic where the loss is very low in the early stages of training. This is because, at the beginning, the model encounters relatively simple positions, allowing the network to fit these scenarios quickly. However, as the model advances and encounters new positions, the network must readjust the distribution of its parameters, causing the loss to increase temporarily before declining again after some time. This cycle continues until reaching step one, at which point the loss stabilizes. In contrast, AlphaZero maintains a more stable downward trend in its loss. The policy loss curve for AlphaZero is relatively flat, while the value loss curve fluctuates significantly. Conversely, BoxesZero experiences greater fluctuations in the policy loss curve but achieves a smoother and faster convergence in the value loss curve. This indicates that BoxesZero places more emphasis on training the value function, whereas AlphaZero focuses more on the convergence of the policy function. The overall downward trend of the curves demonstrates that our model is effective. Moreover, the behavior of the curves explains why our model can achieve strong playing strength more rapidly.

### 6.4. Ablation Studies

To validate the effectiveness of each acceleration strategy, we conducted an ablation study by decomposing our three core methods: the endgame theorem, chain-loop pruning in MCTS, and data augmentation. We assessed their individual impact on overall model performance by comparing the relative ELO ratings of four groups trained under identical parameters (30 h total training time, with models saved at 30 time steps). **Baseline:** Utilizes the endgame theorem, chain-loop pruning and data augmentation simultaneously. **No Endgame:** Removes the endgame theorem from the baseline. **No Chain Loop:** Removes chain-loop pruning from the baseline. **No Data Augmentation:** Removes data augmentation from the baseline.

**Figure 10:**
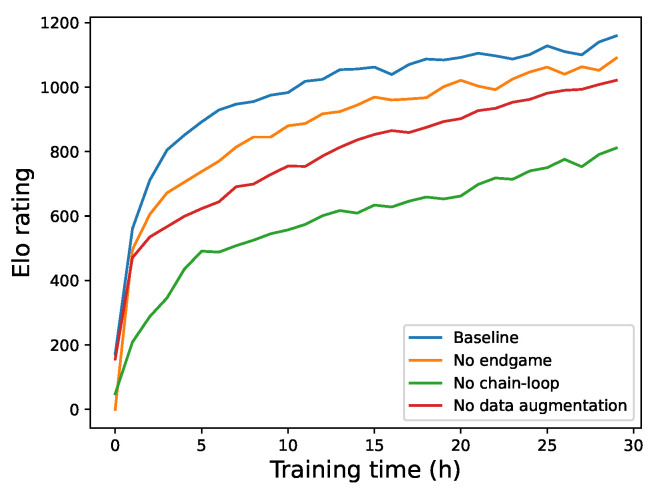
Curves of relative ELO ratings resulting from the ablation study.

We played 1000 games between every pair of models and presented the resulting relative ELO curves in Figure 10. To further analyze performance, we used the models saved at 10, 20, and 30 h to predict outcomes on the datasets described in Section 6.1.2, with the accuracies presented in Table 3. This table highlights the predictive capabilities of the different models at various training durations. The results demonstrate the effectiveness of both our pruning methods and data augmentation.

As shown in Figure 10, the “No Chain Loop” group exhibits the lowest relative ELO score (around 1100 points), indicating that chain-loop pruning provides the most significant performance improvement. Data augmentation follows, with the endgame theorem having the least impact.

Furthermore, the figure reveals synergistic effects between our methods. The combination of the endgame theorem and chain-loop pruning yields notably better results than either method alone. This is likely because chain-loop pruning leads to more reasonable game positions, increasing the likelihood of encountering endgame scenarios and, thereby, amplifying the impact of the endgame theorem. While the endgame theorem’s effect may be relatively minor on a 6 × 6 board, its significance will likely increase on larger boards due to the formation of longer chains and loops.

### 6.5. Evaluation of Model’s Predictive Capabilities at Different Steps

We evaluate the algorithms of BoxesZero and AlphaZero at multiple time points using datasets described in Section 6.1.2. We also include additional random moves for testing. The corresponding prediction accuracies are provided, as shown in Table 4. The orange-marked data in the table indicate the value of parameter st for BoxesZero at the respective current time. For instance, when training reaches 10 h, the parameter st=26, which indicates that training has progressed to step 26.

Through this table, we can clearly see that BoxesZero continues to improve while not forgetting previous knowledge. This is because the old knowledge is reinforced during the advancement, leading to a continuous increase in prediction accuracy.

Specifically, BoxesZero’s prediction accuracy is much higher than that of AlphaZero, especially when training reaches 60 h and 90 h, where the accuracy reached 100%. This reflects the advantages of backward training, as it can fully utilize the real returns to achieve high-level performance with low computational resources.

### 6.6. Analysis of BoxesZero and AlphaZero Data Distributions

**Figure 11:**
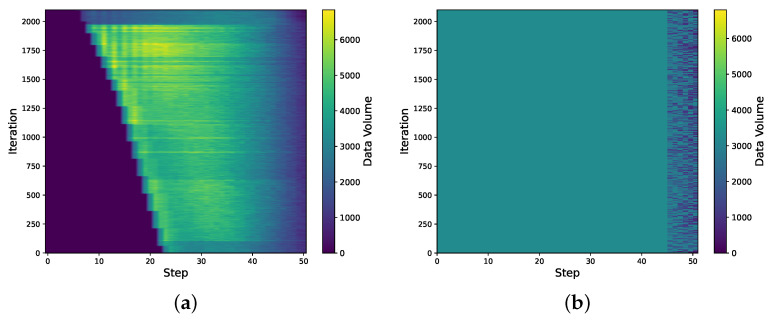
Figure (**a**,**b**) respectively show the heatmaps of the data volume in the experience replay buffer for BoxesZero and AlphaZero at different iterations and steps.

**Figure 12:**
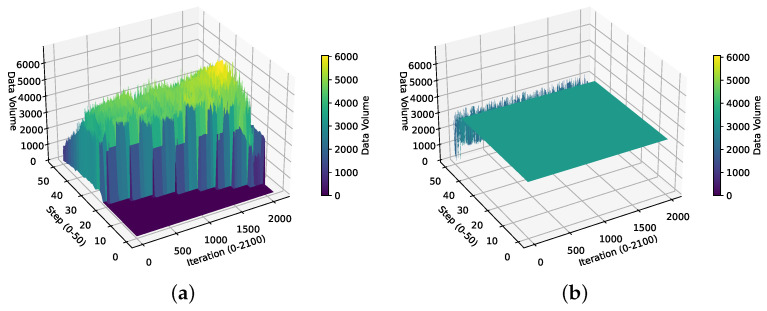
Figure (**a**,**b**) respectively show the 3D surface plots of the data volume in the experience replay buffer for BoxesZero and AlphaZero at different iterations and steps.

We analyze the contribution of Data Augmentation to the data volume and the characteristics of data generated by backward self-play. We present heatmaps of the data volume of BoxesZero and AlphaZero under different steps and iterations, as shown in Figure 11, and 3D surface plots, as shown in Figure 12. This allows us to visually observe the contribution of our data augmentation method to the training data volume, as well as the data distribution characteristics of backward self-play and forward self-play.

Specifically, from the data heatmap of BoxesZero in Figure 11, we can see the obvious data distribution characteristics of BoxesZero. At the beginning, there is only data after st steps. As the iteration increases, the data distribution gradually moves forward. The data heatmap of AlphaZero is almost the same before 40 steps, which is its characteristic: it uses the same amount of data for each step. After 40 steps, the data distribution is less, possibly because one side has already reached 13 points (there are 25 boxes in the 6×6 Boxes game) and won, and no more data are generated. From the color depth of the two heatmaps, we can see that the data volume of BoxesZero is larger than that of AlphaZero, which largely alleviates the problem of data scarcity in reinforcement learning. Moreover, the data volume of Boxes in the earlier steps is larger than that in the later steps, showing a decreasing trend. This reflects that backward training focuses on learning newly encountered situations in each step, and fewer situations are used for review in later steps, which can improve the learning efficiency of the model. Figure 12 analyzes the data distribution from a three-dimensional perspective, and the data advantage of BoxesZero can be clearly seen from the z-axis.

Overall, our method alleviates the problem of data scarcity in reinforcement learning to a certain extent.

### 6.7. Final Performance

To evaluate the final playing strength of BoxesZero, we set the training parameters as illustrated in Table A1. The advance parameters are presented in Table 2. Ultimately, our model utilized a distributed training framework that ran for 10 days, conducted 60,000 self-play games, and incorporated rotation symmetry and data augmentation methods. The feature extraction layer of the neural network consists of 6 parallel blocks with approximately 3 million parameters. In the MCTS, we integrated endgame theorems and employed chain-loop pruning techniques.

**Figure 13:**
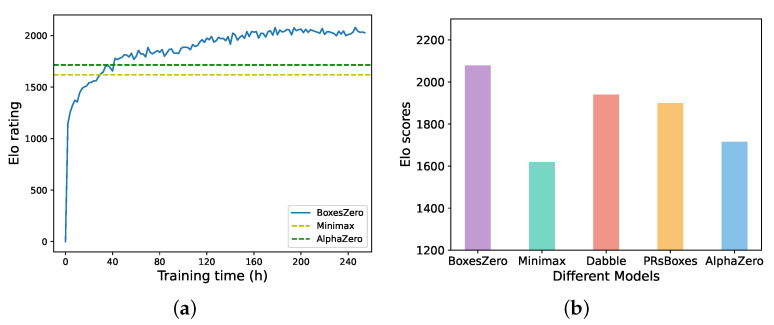
Figure (**a**) illustrates the evolution of the ELO rating for BoxesZero over 240 h of training. Figure (**b**) displays the relative ELO scores obtained by the final model against various open-source models in PK matches.

We evaluated our trained model using the baseline model (see Section 6.1.1). We conducted matches for each pair of models and calculated the corresponding relative ELO ratings. The results are presented in Figure 13. Observing the results, we note that our model was able to defeat the strongest open-source agent Dabble’s algorithm at 130 h of training (st=19). At 110 h of training (st=20), it surpassed the PRBboxes algorithm. Additionally, during the 40 to 50-hour training period, it outperformed both the MiniMax algorithm and the AlphaZero agent trained for 240 h. This indicates that BoxesZero’s playing strength improves faster. Moreover, it is crucial to emphasize that under the aforementioned configuration and after 240 h of training, AlphaZero’s Elo score is indeed lower than that of traditional search algorithms such as Dabble. However, with increased training duration and computational resources, AlphaZero’s performance ceiling will surpass that of traditional search algorithms. Regarding the performance ceiling of BoxesZero and AlphaZero, we believe there is no significant difference between them. This is because when BoxesZero’s backward self-play enters stage 2, its only distinction from the AlphaZero framework lies in the different labels of the value network, which are 0.75z+0.25Q and *z*, respectively.

Finally, upon reaching step 0, we reinforced the policy training. After 15 days of training, we developed our strongest version. Our program ultimately won the championship in the Dots-and-Boxes category of the 2024 Chinese Computer Game Competition.

## 7. Conclusions

We present BoxesZero, an efficient and computationally frugal Dots-and-Boxes agent. We employ a novel backward training approach and extract high-quality nodes within the MCTS process, effectively utilizing rewards and computational resources while mitigating the issue of sparse rewards. Furthermore, we leverage the domain knowledge of Dots-and-Boxes to prune the MCTS, accelerating the convergence of the neural network. Concurrently, we provide and prove endgame theorems encompassing 1-chain and 2-chain scenarios, which were previously unproven. By combining this innovative training method with a domain knowledge-pruned MCTS, BoxesZero achieves strong performance with low computational requirements.

Experimental results demonstrate that BoxesZero requires less time and computational resources than AlphaZero to achieve the same level of performance, and it outperformed the strongest open-source programs available online, including Dabble and PRsboxes. Ultimately, we integrated BoxesZero with the endgame theorems to achieve a championship in the Dots-and-Boxes category of the 2024 Chinese Computer Game Competition.

In summary, we showcase the advantages of backward training, namely its full utilization of rewards and reduction of unnecessary computational resource waste. This architecture can also be transferred to other games, enabling anyone to train a powerful agent using their own computer. Moreover, we contribute to the game of Dots-and-Boxes by completing its endgame theorems, filling a previous gap in the field. Furthermore, we seamlessly integrate domain knowledge with MCTS, enhancing the balance between exploration and exploitation.

However, our model also has limitations. For instance, the control conditions for model advancement are manually set based on repeated observations of the training process, which presents challenges for parameter tuning. Finally, we hope that our model will inspire other areas of artificial intelligence and anticipate further validation of its performance in other reinforcement learning environments.

## Figures and Tables

**Table 1:** Definitions of functions used in endgame theorems. Detailed explanations of the functions’ definitions and computational methods can be found in [23]. In this context, understanding their application is all that is necessary.

Function	Description
size(G)	Number of boxes not yet claimed
θ(G)	Number of 3-chains
f(G)	Number of 4-loops
s(G)	Number of 6-loops
v(G)	Optimal value
c(G)	Controlled value
tb(G)	Terminal bonus

**Table 2:** Model advancement parameters. “—” in the Value column indicates that the parameter is a statistical value and does not require pre-setting.

Parameter	Description	Value
*mqth*	MCTS *Q* threshold at the root node, used to assess the overall capability of the network.	0.85
*nvth*	Network *v* threshold is used to evaluate the network’s predictive ability.	0.75
*nvr*	The ratio of the number of game states in self-play that satisfy the following conditions to the total number of states: $\left|v\right|\geq nvth$ and v×Q≥0.	—
*mqr*	The ratio of the number of game states in self-play that satisfy the following conditions to the total number of states: $\left|Q\right|>mqth$.	—
*mqrth*	The required value of *mqr* for the model to advance.	0.75
*nvrth*	The required value of *nvr* for the model to advance.	0.85
*mit*	The maximum iteration limit per step.	150
β	The number of steps the model advances each time.	1

**Table 3:** This table presents the prediction accuracy of five groups (four ablation study groups and a random group) on datasets with 24–30 steps, under 10, 20, and 30 h training durations. The highest accuracy for each row is highlighted in orange.

Step	Full Component			No Endgame Theorem			No Dada Augmentation			No Chain Loop			Random
(1 K Games)	10 (h)	20 (h)	30 (h)	10 (h)	20 (h)	30 (h)	10 (h)	20 (h)	30 (h)	10 (h)	20 (h)	30 (h)	
24	0.570	0.675	0.701	0.437	0.554	0.695	0.374	0.486	0.620	0.355	0.391	0.435	0.112
25	0.744	0.785	0.795	0.615	0.671	0.792	0.471	0.665	0.760	0.410	0.425	0.545	0.134
26	0.793	0.844	0.872	0.783	0.840	0.865	0.575	0.755	0.765	0.445	0.512	0.610	0.151
27	0.876	0.891	0.912	0.865	0.875	0.901	0.755	0.850	0.900	0.565	0.690	0.821	0.170
28	0.930	0.971	0.979	0.924	0.933	0.934	0.851	0.925	0.940	0.615	0.840	0.904	0.215
29	0.971	0.974	0.987	0.965	0.972	0.980	0.925	0.955	0.971	0.832	0.941	0.952	0.263
30	0.980	0.987	0.992	0.973	0.985	0.990	0.954	0.965	0.989	0.931	0.960	0.972	0.291

**Table 4:** This table presents the prediction accuracy of BoxesZero and AlphaZero on datasets with (22,24,26,28,30) steps under 10, 30, 60, and 90-hour training times. Each column corresponds to a training time, with orange cells indicating BoxesZero’s accuracy at the search’s starting step (st) for that duration.

Step (1 K Games)	BoxesZero (6b)				AlphaZero (6b)				Random
	10 (h)	30 (h)	60 (h)	90 (h)	10 (h)	30 (h)	60 (h)	90 (h)	
22	0.407	0.453	0.645	0.671	0.315	0.355	0.365	0.37	0.115
24	0.475	0.64	0.72	0.745	0.355	0.365	0.38	0.385	0.125
26	0.67	0.83	0.85	0.88	0.38	0.397	0.405	0.425	0.13
28	0.923	0.953	0.971	0.975	0.533	0.532	0.605	0.635	0.217
30	0.984	0.995	1.0	1.0	0.75	0.778	0.82	0.834	0.29

**Figure A1:**
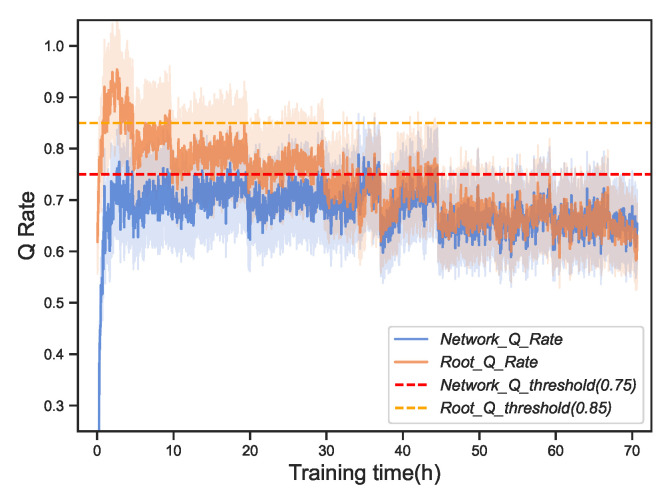
The evolution of the *Q* rate during training. As training progresses, the *Q* values obtained from MCTS gradually approach the state value *Q* predicted by the neural network.

**Figure A2:**
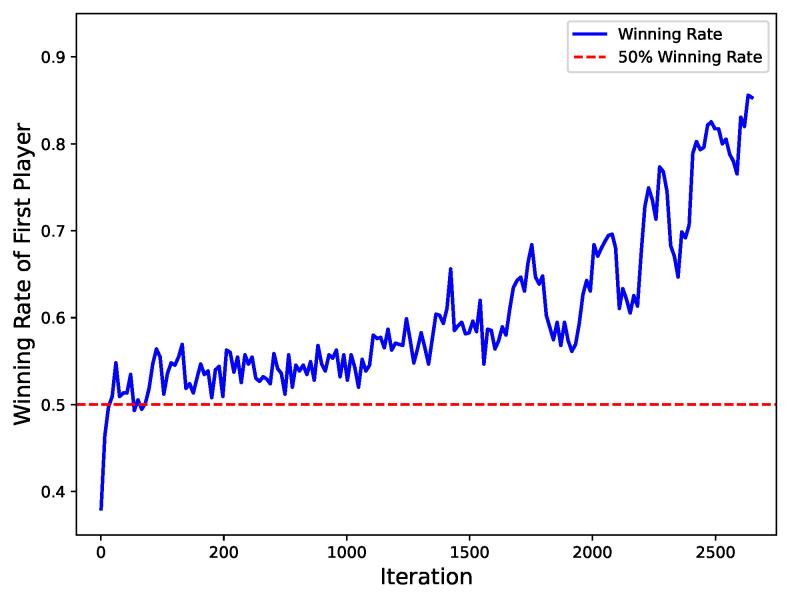
Winning rate of first player over iterations.

**Figure A3:**
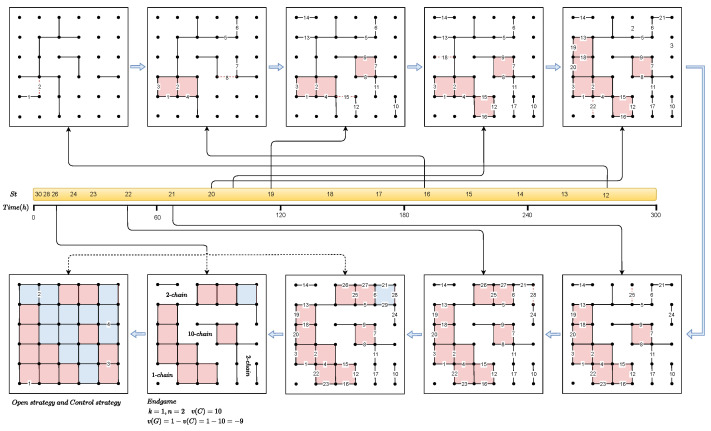
Knowledge acquired by BoxesZero. The horizontal axis at the center represents the training timeline, where the board states indicate the open strategies learned at different times. The final value v=−9 indicates that the blue side can gain 9 additional squares to reach a critical score in the current game situation, utilizing the endgame theorem. st is the parameter for initiating MCTS search in BoxesZero.

## Data Availability

The data presented in this study are available on request from the corresponding author. The data are not publicly available because the research data are confidential.

## Appendix A. Analysis of Q Rate

In order to better understand backward training, we conducted an in-depth analysis of the internal indicators of the model, specifically Q_rate, which refers to the proportion of state values that meet certain conditions. Figure A1 shows the curves of Root_Q_Rate(mqr) and Network_Q_Rate(nvr) over time during the training process. Next, we will analyze this in conjunction with the characteristics of the model.

First, our model begins training from step 30, at which point it encounters positions that are relatively close to the endgame and the complexity of these positions is also relatively low. The root Root_Q_Rate can reach around 0.9, indicating that the return values from MCTS are quite accurate and can clearly reflect the outcome of the positions.

As the model progresses, the Q_Rate exhibits a trend of initially decreasing and then increasing, with the moments of decline corresponding to the periods of model advancement. We can easily conclude that the increase in Q_Rate over time indicates that rewards can be smoothly propagated forward. Moreover, the Q_Rate of the neural network overlaps almost completely after 30 h, suggesting that the predictions of the neural network gradually fit the more accurate *Q* values obtained from MCTS searches, which is key to the success of our backward training. Finally, the Q_Rate stabilizes at around 0.6, which is normal. As the model advances, the amount of data the neural network needs to fit also increases, making it challenging to maintain a consistent level of 0.9.

## Appendix B. Winning Rate Analysis

We present the curve showing how the winning rate of the first player changes with the increase in training iterations see Figure A2 to further our understanding of the backward model. It can be observed that the curve generally trends upward but experiences sudden drops at certain intervals. These troughs correspond to the model’s progress, indicating that when the model encounters new situations, it causes a shift in the network distribution, leading to instability in both the policy and value network. However, as the training progresses, the model gradually converges, ultimately resulting in a winning rate that approaches 1. This characteristic also suggests that the first player holds a certain advantage in the 6×6 Dots-and-Boxes.

## Appendix C. Proof of Extended Endgame Theorem

Based on the previous endgame theorem, we employed mathematical induction to prove the strategies involving 1-chains and 2-chains, along with the corresponding value function v(G). Additionally, we provided open strategies for positions that include both 1-chains and 2-chains.

We first proved the cases involving only 1-chains and only 2-chains, and then based on these proofs, we established the case where both 1-chains and 2-chains are present. v(G;C) represents the margin by which the controller will defeat the opener if the current opener chooses to open C. Therefore, we have the following formula:

(A1) $$v\left(G;C\right)=\left\{\begin{array}{cc} \left(\mathrm{size}(C)-2\right)+\left|v(G-C)-2\right|, & \mathrm{if}\;C\;\mathrm{is}\;a\;\mathrm{chain}\\ \left(\mathrm{size}(C)-4\right)+\left|v(G-C)-4\right|, & \mathrm{if}\;C\;\mathrm{is}\;a\;\mathrm{loop} \end{array}\right.$$

Now consider the opener’s perspective when facing a non-empty position *G*. He will obviously prefer to open whichever component *C* minimizes v(G;C). Therefore, we have the following formula:

(A2) $$v\left(G\right)=\min_{C}v\left(G;C\right)$$

First, we prove Theorem 4 using mathematical induction.

**Proof.** We define *x* as any component other than a 1-chain and 2-chain, *o* as the number of 1-chain, and *t* as the number of 2-chain. G−o−t denotes the part that remains after removing all the 1-chains and 2-chains. **Step 1 (Base Case):** We begin by proving the case where there is only one 1-chain. Since v(G−x)≤v(G−x;1)=1−v(G−x−1)≤1<2, and according to Theorem 2, when v(G−x)<2, the controller should give up control, so when there is only one 1-chain, v(G;x)=x−v(G−x). By Formula (A2), we have v(G;c)≤v(G), and thus,

v(G−x)≤v(G−x;1)=1−v(G−x−1)

Multiplying both sides of this inequality by −1 and then adding *x*, we obtain the following:

x−v(G−x)≥v(G−x−1)+x−1

Since x≥3 and v(G−x−1)≥0 (due to Theorem 3), we obtain the following:

v(G−x−1)+x−1≥2

Furthermore, since v(G−1)≥0, we have v(G;1)=1−v(G−1)≤1. Combining these inequalities, we conclude the following:

v(G;x)=x−v(G−x)≥v(G−1−x)+x−1≥2≥1≥v(G;1)

Thus, v=min(v(G;x),v(G;1))=v(G;1), which means that opening the 1-chain is the optimal move. This completes the proof for the base case. **Step 2 (Inductive Hypothesis):** Suppose the conclusion holds for *k* 1-chains. In other words, when there are *k* 1-chains, opening a 1-chain is the optimal strategy. **Step 3 (Inductive Step):** Now, we prove that the theorem holds when there are k+1 1-chains. When k+1 is even, regardless of when we open the first 1-chain, the next move will also open a 1-chain for the remaining state containing *k* 1-chains. If we open one 1-chain, all of the 1-chains will eventually open. It is known that opening an even number of 1-chains consecutively is equivalent to doing nothing since both players will gain an equal number of squares, and the player order remains unchanged. Therefore, the optimal strategy is to open a 1-chain. When k+1 is odd, the remaining 1-chains will also open if we open the first 1-chain. It is known that opening an odd number of 1-chains consecutively is equivalent to opening one 1-chain. Thus, when there are k+1 1-chains, the proof is the same as for the case with 1 single 1-chain. Therefore, the optimal strategy is to open a 1-chain. By the principle of mathematical induction, the assumption holds for k+1 1-chains. This completes the proof. □

Now, let us proceed to prove the case where only 2-chains are present. The proof follows a similar process to the case involving only 1-chains.

**Proof.** **Step 1: Base Case:** Proving the case for t=1 and o=0. For t=1, we have the following:

(A3) v(G;2)=2−v(G−2)

Since v(G−x)≤v(G−x;2)=2−v(G−x−2)≤2, and according to Formula (A1), if v(G−x)<2, the controller should give up control. Therefore, in the case where t=1,

(A4) v(G;x)=x−v(G−x)

Also, since v(G−x)≤v(G−x;2)=2−v(G−x−2), multiplying both sides by −1 and adding *x*, we obtain the following:

(A5) v(G;x)=x−v(G−x)≥v(G−x−2)+x−2

Now, consider two cases:

- **If *x* is a loop**, we have x≥4. Therefore, from Equation (A5), it is clear that v(G;x)≥2. Since v(G;2)≤2, we can conclude that v(G;2)≤v(G;x), and thus, opening a 2-chain is optimal.
- **If *x* is a chain**, then x≥3. From Equation (A5), we have v(G;2)≤2 and v(G;x)=x−v(G−x)≥1.

Now, we proceed by contradiction. Assume that there exists a situation where v(G;2)>v(G;x). Then, we must have v(G;2)=2 and v(G;x)=x−v(G−x)=1. From this it follows that v(G−2)=2−v(G;2)=0 and v(G−x−2)+x−2=1. Using Equation (A1), we know that v(G-2;x) = x − 2 + |v(G-x-2) − 2|. Substituting this into the previous equation gives v(G−2;x)=2x−3. According to Lemma 2.2, we know that v(G−2)≡v(G−2;x)(mod2). However, v(G−2)=0 and v(G−2;x)=2x−3 are odd, so they cannot be congruent, which leads to a contradiction. Therefore, we conclude that there is no case where v(G;2)>v(G;x). Hence, v(G)=v(G;2), and opening the 2-chain is optimal. This completes the proof. **Step 2: Inductive Hypothesis:** Assume the conclusion holds for t=k (i.e., for *k* 2-chains). **Step 3: Inductive Step:** Next, we prove the case for t=k+1.

- If k+1 is even, the proof is similar to the case for 1-chains, and it is evident that opening a 2-chain is optimal.
- If k+1 is odd, the proof is analogous to the case for t=1, and opening a 2-chain is optimal.

Therefore, the conclusion holds for t=k+1. This completes the proof. □

Finally, we prove the case where both 1-chains and 2-chains are present.

**Proof.** **Step 1: Base case:** Proving the case for o=1 and t=1. From the previous proofs for the cases (o=0,t=1) and (o=1,t=0), we know the following: If the 1-chain is opened first, the next move must open the 2-chain. Therefore, we have

v(G;1)=v(G−1−2)−1

If the 2-chain is opened first, the next move must open the 1-chain, giving

v(G;2)=v(G−1−2)+1

Since the 1-chain and 2-chain are opened consecutively, and o+t is even, we can treat this combination of 1-chain and 2-chain as a single component. Within this component, opening the 1-chain first is optimal as the opener can score 2 points, while the opponent only gains 1 point, maximizing the net score difference. Thus, whoever claims this component will net 1 point without changing the player turn. Clearly, the optimal strategy is to first open the 1-chain, followed by the 2-chain, and then continue with the remaining *x*. Q.E.D. Proving the case for o=1 and t=2. Building on the proof for (o=1,t=1), these three parts can be again viewed as a single component. Within this component, the optimal order is to open the 1-chain first, followed by both 2-chains. This results in the player scoring 2 points while the opponent scores 3 points, with the opponent gaining a net 1 point and the turn switching to the opponent. This effect is equivalent to the value of a single 1-chain: awarding 1 point to the opponent and switching the player. We have already proven the optimal strategy for the case o=1. Thus, the optimal strategy in this case is to first open the 1-chain, followed by the 2-chains, and then open the remaining *x*. Q.E.D. **Step 2: Inductive Hypothesis:** Assume the conclusion holds for o+t=k. **Step 3: Inductive Step:** We now prove the case for o+t=k+1. Let the newly added component be *C*. Regardless of whether *C* is a 1-chain or a 2-chain, once we open *C*, the remaining o+t=k case will follow the optimal strategy of opening a sequence of 1-chains and 2-chains. We can still treat the entire sequence of 1-chains and 2-chains as a single component, where adjacent pairs of identical components cancel out because they do not change the player’s turn or score. After canceling out even pairs, the remaining sequence will have one of three forms:

C1,C2,C12

Substituting *C* for a 1-chain or a 2-chain, we obtain six possible cases:

11,21,12,22,112,212

Using the conclusions of the previous steps, we deduce that the optimal strategy in all cases is to first open the 1-chain, followed by the 2-chain, and then proceed with the remaining *x*. Thus, we have proven that for any endgame involving an arbitrary number of 1-chains and 2-chains, the optimal strategy is to open the 1-chains first, then the 2-chains, and finally the remaining *x*. Q.E.D. Next, based on the opening strategy, we can merge the sequences of 1-chains and 2-chains, cancel adjacent pairs of identical components, and simplify the sequence. The simplified sequence can then be used to determine the value v(G).

- If *o* is odd and *t* is odd, the simplified sequence is [1,2], o+t is even, and the player does not change. Thus, v(G)=v(G−o−t)−1.
- If *o* is odd and *t* is even, the simplified sequence is [1], o+t is odd, and the player changes. Therefore, v(G)=1−v(G−o−t).
- If *o* is even and *t* is odd, the simplified sequence is [2], o+t is odd, and the player changes. Hence, v(G)=2−v(G−o−t).
- If *o* is even and *t* is even, the simplified sequence is [None], o+t is even, and the player does not change. Thus, v(G)=v(G−o−t).

□

## Appendix D. From Foolishness to Success: Turning Points in BoxesZero’s Gameplay

By observing the training process of BoxesZero, we noted some moves that defy human intuition. These seemingly foolish strategies yielded surprisingly good results. Advanced players of Dots-and-Boxes typically avoid playing the third edge of a box in the early game to prevent giving their opponent a box. However, BoxesZero sometimes chooses to play the third edge even when there are many options available that do not yield a box during the early and midgame. We can only explain this phenomenon by suggesting that giving away the box allows for an exchange of turn order, enabling control of advantageous positions in the later stages of the game. We present a replay of a game that defies intuition, along with the time points when this strategy was learned and our model’s parameter St. As shown in Figure A3, the blue player gave away 9 boxes during the early and midgame but ultimately won the match as the controller of a valuable endgame worth 9 points, finishing with a 1-point advantage.

## Appendix E. Details of Training

In this subsection, we will present the specific training details for BoxesZero and AlphaZero used in the experiments. The parameters were determined through a series of experiments exploring different parameter combinations and iterative performance optimization.

### Appendix E.1. AlphaZero Training Details

The training parameters for AlphaZero in Boxes are presented in Table A1.

**Table A1:** BoxesZero and AlphaZero network training hyperparameters.

Hyperparameter	Description	Value
D	Number of recent iterations stored in the replay buffer.	5
γ	Data decay factor	0.98
lr	Learning rate	0.001
epochs	Number of epochs per batch	5
batch size	Batch size for training	1024
N	MCTS search nodes	800
Ngame	Number of self-play games in one iteration	50
$C_{\mathrm{puct}}$	Exploration constant	2

The neural network structure of AlphaZero is the same as that of BoxesZero, with both using a 3-channel input layer and 6 parallel blocks in the feature extraction layer. The loss function consists of three main components: policy loss, value loss, and regularization loss. Their specific forms are as follows:

We utilize an experience replay pool to retain all data generated from the last 5 iterations for sampling training, and we limit the total number of search nodes for each MCTS tree. The following is the loss function of AlphaZero:

- **Policy Loss**: This is used to measure the difference between the predicted policy and the true policy, which is represented by cross-entropy loss, as shown in the following equation:
  (A6) $$L_{\mathrm{policy}}=-\sum_{i\in \mathrm{moves}}\pi_{i}\log\left({\hat{p}}_{i}\right)$$
  where $\pi_{i}$ is the true policy distribution, and ${\hat{p}}_{i}$ is the model’s output policy distribution.
- **Value Loss**: This is used to evaluate the accuracy of the model’s prediction of the value of the state, which is represented by the mean squared error, as shown in the following equation:
  (A7) $$L_{\mathrm{value}}={\left(z-\hat{v}\right)}^{2}$$
  where *z* is the actual value of the state, and $\hat{v}$ is the value predicted by the model.
- **Regularization Loss**: This is used to prevent overfitting the model, which is typically represented by L2 regularization, as shown in the following equation:
  (A8) $$L_{\mathrm{reg}}=\lambda {∥\theta ∥}^{2}$$
  where λ is the regularization parameter and θ are the weights of the model.
- **Total Loss Function**: Combining the above three components, the total loss function for AlphaZero can be expressed as follows:
  (A9) $$L_{\mathrm{total}}=L_{\mathrm{policy}}+L_{\mathrm{value}}+L_{\mathrm{reg}}$$

### Appendix E.2. BoxesZero Training Details

The training parameters for BoxesZero are shown in Table A1. Additionally, we present the parameter settings for the advancement conditions of the BoxesZero model in Table 2, as well as the parameter settings for data augmentation in Table A2. BoxesZero uses the loss function in Equation (6).

**Table A2:** Data augmentation constraint parameters.

Step Range	$N_{\mathrm{threshold}}$ (×N)	$Q_{\mathrm{threshold}}$
st≤step<st+3	0.15	0.5
st+3≤step<st+5	0.2	0.6
st+5≤step<st+10	0.21	0.7
st+10≤step<st+15	0.22	0.8
st+15≤step	0.23	0.85

### Appendix E.3. Dataset Construction Methods

We replicated the alpha-beta search and several pruning strategies mentioned in Barker [20], and combined them with the endgame theorem presented in this paper. This allowed us to search for and find winning positions within approximately 22 steps. We utilized this method to generate a set of winning states, saved as (state, winning move list) pairs. This enabled us to create datasets for various game steps, ranging from 22 to 30, with each step containing 1000 positions.

We used different network parameters θ combined with MCTS to search each position in the datasets for 5 s. We then checked if the move returned by MCTS existed within the corresponding winning move list. If it did, the prediction was considered correct.

Finally, we calculated the prediction accuracy for each step’s dataset to compare the accuracy of different models at different game stages.
